# Microwave scan and brain biomarkers to rule out intracranial hemorrhage: study protocol of a planned prospective study (MBI01)

**DOI:** 10.1007/s00068-021-01671-7

**Published:** 2021-05-04

**Authors:** Tomas Vedin, Henrik Bergenfeldt, Emanuel Holmström, Jakob Lundager-Forberg, Marcus Edelhamre

**Affiliations:** Clinical Sciences, Lund University, Svartbrödragränden 3-5, 251 87 Helsingborg, Sweden

**Keywords:** TBI traumatic brain injury, Biomarkers, Microwaves, CT scan, X-ray, Brain hemorrhage, traumatic

## Abstract

**Purpose:**

The aim of this planned study is to evaluate the ability of a cranial microwave scanner in conjunction with nine brain biomarkers (Aβ40, Aβ42, GFAP, H-FABP, S100B, NF-L, NSE, UCH-L1 and IL-10) to detect and rule out traumatic intracranial hemorrhage in an emergency department setting. Traumatic brain injury is a world-wide topic of interest for researchers and clinicians. It affects 2% of the population per annum and presents challenges for physicians as patients’ initial signs and symptoms do not always correlate with the extent of brain injury. The gold standard for diagnosis of intracranial hemorrhage is head computerized tomography (CT) with the drawbacks of high cost and radiation exposure. A fast, secure way of diagnosing without these drawbacks has potential to make care more effective and reduce cost.

**Methods:**

Study will be prospective and enroll adult, consenting patients with head trauma who seek emergency department care. Only patients where the treating physician prescribes a head-CT will be included. The microwave scan and blood sampling will be performed in close temporal proximity to the CT scan. Results will be analyzed with sensitivity, specificity and receiver operator characteristics analysis to provide the best combination of a number of biomarkers and the microwave scan.

**Conclusion:**

This study will explore the diagnostic accuracy of a head microwave scanner in combination with biomarkers in ruling out intracranial hemorrhage in traumatic brain injury patients presenting to the emergency department. Potentially, this combined diagnostic approach could achieve both high sensitivity and high specificity, thereby reducing the need of CT-head scans when managing these patients.

Clinicaltrials.gov identifier: NCT04666766. Registered December 11, 2020.

## Introduction/Background

Traumatic brain injury (TBI) affects 2% of the population per annum and is a major cause of morbidity, mortality, and emergency department (ED) visits [1]. Furthermore, it presents challenges for physicians in the emergency setting, because patients’ initial signs and symptoms do not always correlate with the extent of brain injury [2, 3]. The age of afflicted patients has increased in the past decade and the outcome of elderly patients is often worse, in part because of the widespread use of anticoagulants and thrombocyte inhibitors [4, 5]. It is paramount to detect and treat these injuries as soon as possible, to avoid both unnecessary subsequent damages and casualties because of long transfer times [1]. Under-triage of major trauma has been shown to cause substantial and potentially avoidable damage [6]. Intracranial hemorrhages can be difficult to detect by clinical examination. Even if the physician follows a clinical practice guideline, it can be difficult to know from signs, symptoms and patient history which patient will have an intracranial hemorrhage [7, 8]. It can also be difficult to predict what patients will need neurosurgical intervention [8].

As neurosurgery is regularly only available at tertiary hospitals, most patients are primarily investigated at hospitals without neurosurgery. A faster diagnosis would enable prompt transport of these patients to the correct level of care. Currently, there are no methods of reliable prehospital diagnosis as it requires access to computerized tomography (CT) of the head and a radiologist’s interpretation of the images. The head-CT is gold standard for diagnosing intracranial hemorrhage [9].

Brain biomarkers can be used to assess the risk of traumatic intracranial hemorrhage more accurately. Currently, the biomarker S100B is used in Scandinavia with a sensitivity of up to 99% and a specificity of about 30% [10]. Combination of biomarkers with similar performance has been researched and a combination of two biomarkers is used in the USA [11, 12]. A clinical guideline that incorporates a biomarker ensures that no intracranial hemorrhages requiring neurosurgical intervention are missed. However, this high level of security gives low specificity with many negative CT-scans as a consequence. A study of the Scandinavian Neurotrauma Committee (SNC) Guideline which incorporates serum protein S100B-level showed very good safety, but 67% of the patients underwent a CT scan. Of these scans, 92.5% were negative [13]. In spite of this, a reduction in CT-scans has been seen when applying the SNC Guideline in both North American and Scandinavian settings [7, 13]. Apart from the cost of at least $200/CT scan and prolonged ED stay, there is also the small but significant risk of radiation-induced cancer to take into account [14–16]. To reduce radiation exposure, cost and ED waiting time, the ED management of TBI needs to be further improved. The internal cost of serum S100B protein level assay at our facility is $20 during office hours and $40 out of office hours. Time from blood sampling to laboratory report is around 90–120 min.

## Microwave detection of intracranial hemorrhage

A device (MD100™) that utilizes microwaves to detect intracranial hemorrhages has been developed by Medfield Diagnostics AB, Gothenburg, Sweden. The radiation exposure per scan is far less than that of a cell phone call. The MD100 has been used to detect intracranial hemorrhage in stroke patients [17, 18] and on patients with larger subdural hematomas and planned neurosurgical intervention. In that setting, the sensitivity was 100% and the specificity was 75% [19]. However, smaller hemorrhages and different anatomic sites (e.g. temporobasal hemorrhage) could yield lower detection performance and using an adjunct such as a brain biomarker in combination with the MD100 might raise the detection accuracy. Currently, there is no clinical data investigating the limitations of detecting hemorrhages at more difficult anatomical sites.

There is, to our knowledge, no study on microwave-based scanning for traumatic intracranial hemorrhage in conjunction with brain biomarkers.

## Brain biomarkers

**Fig. 1:**
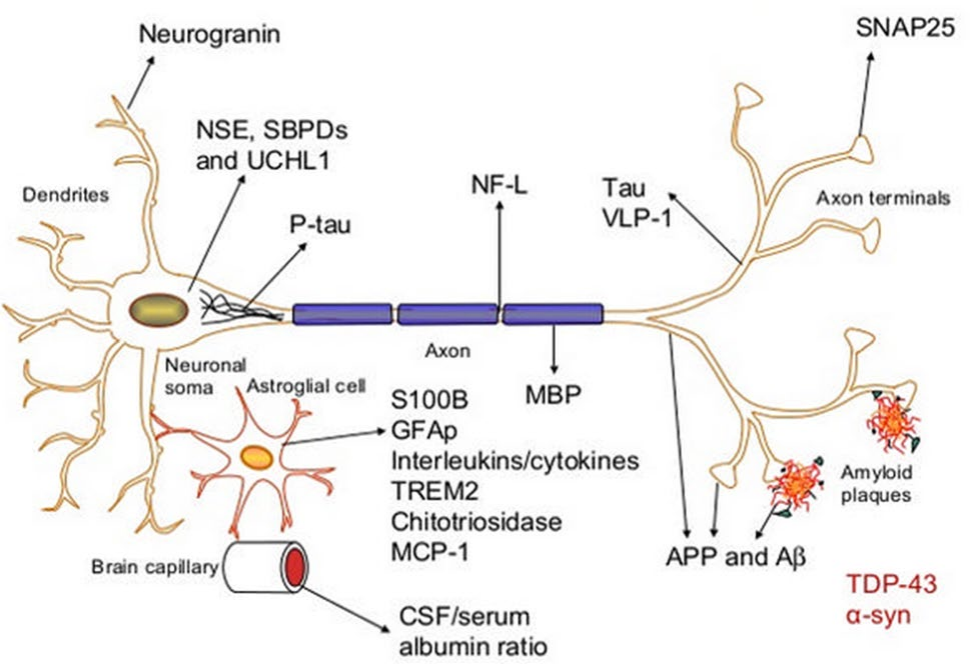
Figure is showing the origin of study biomarkers. Published with permission by creator, Henrik Zetterberg, professor of clinical chemistry, Gothenburg University

During the past decade, several biomarkers have emerged. These include S100 calcium-binding protein β (S100B), Glial fibrillary acidic protein (GFAP), Ubiquitin C-terminal hydrolase L1 (UCH-L1), Neuron-specific enolase (NSE), Tau, Neurofilaments, Myelin sheath marker (MSM), β-amyloid isoforms, Heart fatty-acid-binding protein (H-FABP), Interleukin 10 (IL-10), Alfa-II spectrin breakdown products, etc. [20]. See Fig. 1 for illustration of the origin of the biomarkers.

However, the scientific literature is heterogenous and most studies investigate biomarkers’ respective roles in predicting treatment and outcome of severe TBI. Also, time from trauma to biomarker sampling might vary between 4 and 24 h after trauma and studies do not always discriminate between degrees of TBI. The majority of TBIs are mild (80–90%)[21]. A recent metaanalysis studying biomarker use mild TBI patients concluded that only S100B had sufficient evidence and both research heterogeneity and knowledge gaps were outlined [22]. A current literature review indicated that panels of biomarkers may have better diagnostic performance than any single biomarker [23].

A prospective study of eight biomarkers analyzed both stand-alone and in batteries (β-amyloid isoforms 1–40 (Aβ40) and 1–42 (Aβ42), GFAP, H-FABP, IL-10, neurofilament light (NF-L), S100B, and tau) indicated that high sensitivity of single biomarkers gave low specificity and that panels of biomarkers had high sensitivity and significantly increased specificity. The panel with the best performance (H-FABP, S100B and tau) did not contain the biomarkers with the best performance when analyzed separately [24].

### Selection of biomarkers

Because of the disparity of current brain biomarker research, a large panel of different biomarkers has been chosen for this study:

- Aβ40
- Aβ42
- GFAP
- H-FABP
- S100B
- NF-L
- NSE
- UCH-L1
- IL-10

### Biomarker assay

Within 2 h of sampling, the blood will be centrifuged at 2200*g* for 15 min and aliquoted into serum and plasma tubes. These will be frozen at 70 °C and assays will be performed once a month in a research laboratory at Helsingborg General Hospital. All analyses will be done with enzyme-linked immunosorbent assay (ELISA)-kits intended for research. These ELISA-kits will be validated before using them in the present study.

To enable future research, 4 × 2 ml serum in 40 aliquots will be saved at 70 °C.

## Microwave scanner mechanism of action

The MD100 is non-invasive, portable, rechargeable and certified by the Swedish National Certification Body. Microwave signals are sensitive to dielectric parameters of the investigated object. Brain tissue has different dielectric properties than blood and an intracranial hemorrhage changes cerebral dielectric parameters. These changes are detected as amplitude shifts in the diagnostic frequency interval 0.1–2 GHz.

The MD100 instrument consists of a headrest with eight antennas, an electronic switchbox, a network analyzer and a computer controlling measurements and collecting data. During the measurement, one antenna at a time will send out an electromagnetic signal from 0.1 to 2 GHz where each antenna will act as receiver in sequence. The transmitted power from each antenna will be approximately 0.1 mW. All antennas will act as transmitters and receivers in sequence. The data matrix that is generated from the measurement is analyzed and changes in the dielectric properties are evaluated. An algorithm based on simulations and measurements from actual patients has been established ahead of this clinical study and the algorithm will not change during the study. The MD100 is not self-learning.

### Study aim

The aim of this study is to evaluate the stand-alone diagnostic accuracy of the MD100 and in combination with different brain biomarkers when ruling out intracranial hemorrhage in patients with trauma to the head.

## Methods

### Study design

This study is a single-center prospective cohort study which will start in April 2021 and will run until patient inclusion is finished. This manuscript represents the latest version of the study protocol. The study is approved by the Swedish Ethical Review Government Body and the Medical Products Agency and is registered and Clinicaltrials.gov. Amendments to the protocol will be forwarded to the Swedish Ethical Review Government Body and the Medical Products Agency for notification and approval.

### Study setting

The study will be set at Helsingborg General Hospital, Helsingborg, Sweden. The hospital has a catchment area of 300,000 people and approximately 75,000 ED visits per annum with 1800 isolated TBI patients and another 1000 multi-trauma patients [25]. The SNC Guideline is the hospital recommended guideline. The hospital has 24 h surgeons with sub-training in trauma, emergency medicine specialists, anesthesiologists, orthopedic surgeons and ear–nose–throat doctors but no neurosurgical clinic. The trauma surgeons are trained to perform emergency craniotomies and the closest neurosurgical clinic is 45 km away at Skane University Hospital in Lund. Patients in need of neurosurgical intervention but stable enough for ambulance transport will be moved there.

### Participants

A total of 400 patients will be included in this diagnostic part of the study. Forty of these will have intracranial hemorrhage. As a control group, an additional 50 demographically matched healthy volunteers will be asked to participate in the trial.

The study population will be subsequently divided into three study groups:

- Group A: Patients diagnosed with intracranial hemorrhage by head-CT scan.
- Group B: Patients where intracranial hemorrhage was ruled out by head-CT scan.
- Group C: Healthy volunteers without prior head trauma.

**Fig. 2:**
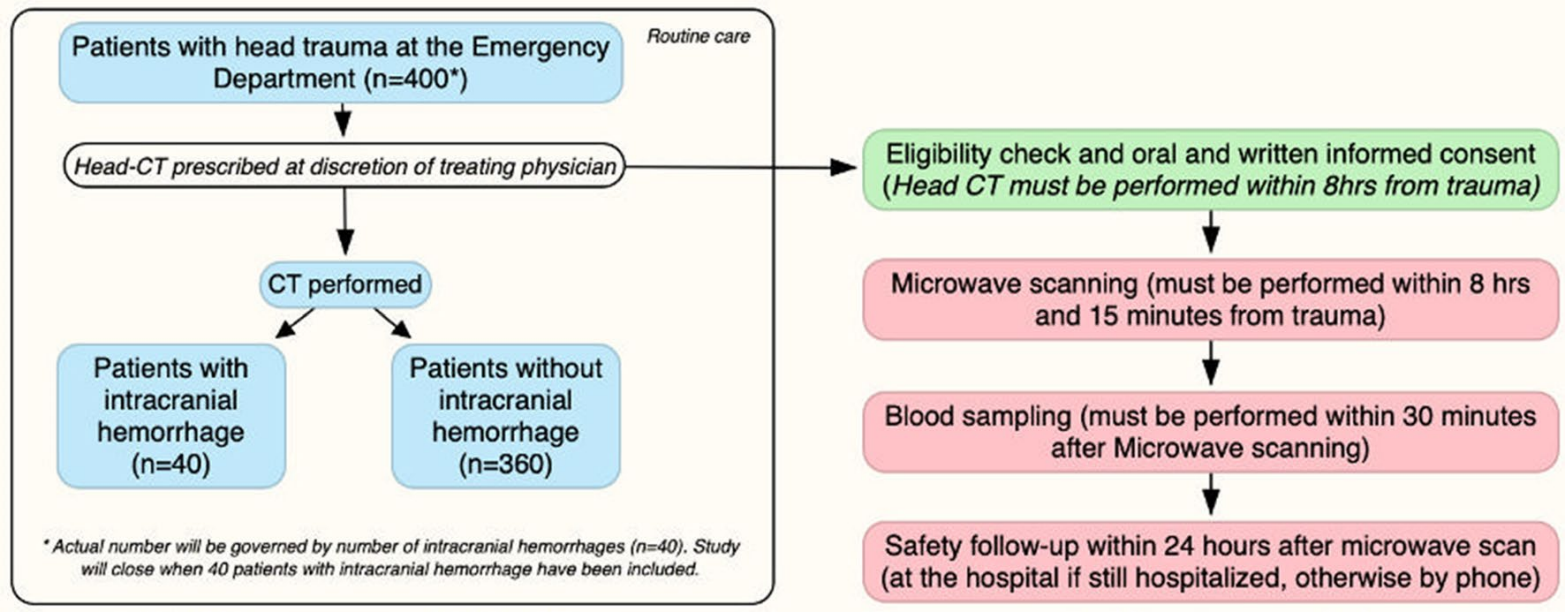
Flow chart of inclusion process for Group A and Group B

See Fig. 2 for flow chart of inclusion process in Group A and Group B.

*Inclusion criteria*, Group A and B (all criteria must be met)

- Verbal informed consent in the acute phase.
- Signed informed consent form after the acute phase. On behalf of the patient, independent witness signed informed consent (in cases where the patient is unable to sign).
- Acute trauma patient with suspected head injury.
- Time from injury to measurement procedure not longer than 8 h 15 min.
- Head CT prescribed by treating physician discretion.
- Patient is ≥18 years of age.
- Patient is deemed clinically stable, per treating physician’s judgment.

*Exclusion criteria*, Group A and B (no criteria can be met)

- Patient has suspected cervical spine fracture, per the investigator’s judgement.
- Patient has by CT confirmed cervical spine fracture.
- Patient has confirmed skull fracture with risk for dislocation.
- Patient has a shunt or other foreign object implanted intracranial (if known by medical records).
- Patient has agraffes or other metal parts, thick bandage (>1 cm), or other foreign materials attached to the head that are deemed to interfere with the diagnostic procedure.
- Patient diagnosed with a condition associated with risk of poor protocol compliance.
- The measurement procedure is deemed to interfere with the standard of care.
- Other condition or symptoms preventing the patient from entering the trial, per the investigator’s judgment.

*Inclusion criteria*, Group C (all criteria must be met)

- Patient is ≥ 18 years of age.
- Signed informed consent.

*Exclusion criteria,* Group C (no criteria can be met)

- Previous stroke or other diagnosed and/or treated brain injury

#### Ethical conduct

The investigation is designed and shall be performed in accordance with the MDD 93/42/EEC Annex X, with the ethical principles that have their origin in the declaration of Helsinki and that are consistent with good clinical practice (GCP) and applicable regulatory requirements. The study will be conducted in compliance with ISO 14155:2011.

### Recruitment

Patients with trauma to the head (isolated or as part of multi-trauma) presenting at the ED at Helsingborg General Hospital will be identified by the nurse-in-charge and the study staff will be alerted. If the physician-in-charge prescribes a head-CT as part of the clinical investigation, he or she will inform the patient briefly about the study and ask about interest to participate. If the patient is interested, a study representative will approach the patient with oral and written information about the study and will acquire oral and written consent if the patient consents to participate.

### Study outcomes

#### Primary outcome

The ability of the device MD100, with and without brain biomarkers (Aβ40, Aβ42, GFAP, H-FABP, S100B, NF-L, NSE, UCH-L1 and IL-10) to detect traumatic intracranial hemorrhage, as measured by the area under the receiver operator characteristics (ROC) curve and confusion matrix.

#### Secondary outcomes

- Assess the accuracy of the device in estimating volume (ml) and location of the intracranial hemorrhage.
- Assess the performance of brain biomarkers, both alone and in conjunction with each other, as measured by area under the receiver operating characteristics curve and confusion matrix.
- Adverse event occurring within 24 h from microwave scanning.
- Assess the practicality and safety of having a bedside scanner in the acute head trauma patient in a triage and trauma ED setting.

### Safety

The safety of MD100 has been evaluated in a human population by Ljungqvist et al. without any severe adverse device events [19]. Because of the small radiation dose each scan delivers, there are no substantial theoretical risks of the scanning process itself. The investigator is responsible for the detection and documentation of adverse events (AEs). The expected AEs are associated with the physical positioning in the device and not the measurement itself. AEs will be collected for the duration of the investigation and recorded in the electronic Case Reference File (eCRF). The AEs will be collected by a member of the staff performing a standardized interview with the patient 24 h after scanning asking for perceived discomfort. It is the responsibility of the investigator to ensure that all information is correct and that all staff involved in the investigation are familiar with the definitions and procedures of AE reporting.

In the case of an AE, the investigator will initiate the appropriate treatment according to their medical judgment. In most cases, this means notifying the patient’s primary health care provider.

In our institution, blood is routinely drawn from TBI patients needing further ED assessment (e.g. head CT or S100B according to current guidelines). Thus, sampling blood for biomarker assay from the patients does not add any additional risk of complications and the patient-specific risks of sampling blood are deemed very low.

To decrease the risk of aggravating cervical spine injuries by manipulating the head, the patients are only included if there is no clinical suspicion of this injury. Previous research has shown that patients with trauma to the head who are Glasgow Coma Scale (GCS) 13–15 do not have an increased risk of cervical spine injury [26]. A previous study by Vedin et al. [7, 21, 25] at Helsingborg General Hospital on a very similar patient cohort has shown that approximately 99% of the patients are GCS 13–15. Hence, the theoretical risk of aggravating a pre-existing cervical spine injury is very low with this study design.

### Data analysis—sample size

We know from previous studies that approximately 5% of ED TBI patients have intracranial hemorrhages [23]. We assumed that the sensitivity of the testing will be 99% (i.e. 99/100 patients testing positive will have an intracranial hemorrhage) and the specificity will be 70% (i.e. 30/100 patients without intracranial hemorrhage will test positive). We choose a precision of 0.05 and factored in a 10% dropout. The sensitivity will be 99% ± 5% at the sample size of 378 patients (including the 10% dropout) and the included number of intracranial hemorrhages needs to be 40. Thus, approximately 400 patients will be included in the study. The exact number of patients will be determined by the number of patients with intracranial hemorrhage (i.e. 40).

### Data analysis—procedures for data checking

Data from the microwave measurement will be transferred to a reference database in encrypted zip files via an HTTPS protocol. Clinical data will be manually entered and stored in an eCRF. The eCRF is compliant with the general data protection regulation and national data management laws. All data processed during the trial will only be identified by patient/subject enrolment number, thereby ensuring that the patient’s identity remains hidden. Any information kept on paper will be locked in and without patient identity. The study will have an external monitor in accordance with GCP guidelines. Statistical processing will be performed with SPSS v. 25.

### Data analysis—statistical analysis

The diagnostic performance of both biomarkers and MD100 in conjunction will be summarized by an area under the curve with standard errors and 95% confidence interval where the best sensitivity and specificity will be highlighted. Descriptive statistics will be calculated for all variables, and distributional assumptions will checked with the proper tests and graphs. Demographics, etc., will be presented in tables, figures, and text. Sensitivity, specificity, negative predictive value, positive predictive value, negative likelihood ratio and positive likelihood ratio for the MD100’s ability to identify traumatic intracranial hemorrhage will be calculated. Sensitivity, specificity, negative predictive value, positive predictive value, negative likelihood ratio and positive likelihood ratio for the individual biomarkers’ and biomarker panels’ ability to identify traumatic intracranial hemorrhage will be calculated. Confusion matrix for the combination of MD100/biomarkers’ ability to identify traumatic intracranial hemorrhage will be performed. The Bonferroni method, or other appropriate correction methods, will be used as needed to adjust the alpha-level of individual statistical tests so that the overall level of statistical significance is not deteriorated when performing multiple statistical analyses. The precisions for estimating the position and volume of the intracranial bleedings will be evaluated using appropriate correlation coefficients between estimated values and values from CT scan. Further statistical methods might be used to fully assess the primary and secondary outcomes. We expect a slight imbalance since some subject will only get a subset of the microwave or bio marker measurements performed due to failure to perform the measurements. For scenarios when this has an impact, proper corrections will be utilized.

## Discussion

The current clinical management of head trauma is rather safe when it comes to detecting patients with intracranial hemorrhages that require intervention [13]. However, this safety comes with the cost of many negative CT-head scans. This is time-consuming, expensive and exposes the patient to potentially harmful radiation [14, 15]. The annual ED influx of head-trauma patients is substantial [7, 21, 25]. Even small improvements in the clinical management that lead to decreased processing time, decreased use of radiation and/or decreased cost due to the use of brain biomarkers and other diagnostic tools, such as microwave machines, would benefit the health care system and patients alike [7, 21, 25].

Currently, there is a knowledge gap in brain biomarker research in the setting of emergency management of TBI and consequently no consensus as to what biomarker(s) should be used and how [27]. This study will provide useful knowledge on how accurate the MD100 is in diagnosing intracranial hemorrhage, both with and without the use of brain biomarkers. Also, it will provide insight in how several, both well- and not so well-researched brain biomarkers can be used stand-alone, in batteries of several biomarkers and in conjunction with the MD100 to distinguish TBI patients with intracranial hemorrhage from TBI patients without intracranial hemorrhage.

This study bares some limitations. First, the evaluation of intracranial hemorrhage performed with the initial head-CT will give information on whether or not there is a hemorrhage at that particular time. Thus, potentially slow-developing hematomas not detected on the first CT could be missed. However, the incidence of delayed intracranial hemorrhage is low (< 0.8%) [28, 29]. Second, diffuse axonal injury and cerebral edema, which are not always visible on the initial head-CT scan, might lead to higher levels of some brain biomarkers [30, 31]. Third, it is not known how these injuries might affect the microwave measurement and this could potentially bias the measurement and without performing other radiography capable of visualizing this, there is no way of correcting for this. Fourth, the exclusion of patients with suspected or diagnosed cervical spine injury will exclude a group of patients who have sustained a trauma of higher energy.

The strengths of the study are the selection of patients that benefit from a management that maintains the diagnostic accuracy but entails fewer head-CTs, the fact that all patients will undergo a head-CT scan so that intracranial hemorrhage can be definitely diagnosed or ruled out, the wide array of biomarkers that have not been tested in this setting, neither as stand-alone predictors or in conjunction with a microwave scan and the inclusion of patients incapable of giving written informed consent at the time of trauma.

We believe that, when validated by more specific studies, a diagnostic tool using one or several brain biomarkers in combination with a microwave measurement will be readily and sustainably implementable in standard care. The diagnostic ability can be explored in the present study. However, possible benefits of using less radiation, expedited management with faster diagnosis and reduced cost are feasible gains but remain to be explored in future studies.

## Conclusion

This study will explore the diagnostic accuracy of a head microwave scanner in combination with biomarkers in ruling out intracranial hemorrhage in TBI patients presenting to the ED. Potentially, this combined diagnostic approach could achieve both high sensitivity and high specificity, thereby reducing the need of CT-head scans in TBI patients.

## Data Availability

Parts of the data will be made available upon request.
